# Computer usage and task-switching during resident’s working day: Disruptive or not?

**DOI:** 10.1371/journal.pone.0172878

**Published:** 2017-02-24

**Authors:** Marie Méan, Antoine Garnier, Nathalie Wenger, Julien Castioni, Gérard Waeber, Pedro Marques-Vidal

**Affiliations:** Division of General Internal Medicine, Lausanne University Hospital, Lausanne, Switzerland; Northwestern University, UNITED STATES

## Abstract

**Background:**

Recent implementation of electronic health records (EHR) has dramatically changed medical ward organization. While residents in general internal medicine use EHR systems half of their working time, whether computer usage impacts residents’ workflow remains uncertain. We aimed to observe the frequency of task-switches occurring during resident’s work and to assess whether computer usage was associated with task-switching.

**Methods:**

In a large Swiss academic university hospital, we conducted, between May 26 and July 24, 2015 a time-motion study to assess how residents in general internal medicine organize their working day.

**Results:**

We observed 49 day and 17 evening shifts of 36 residents, amounting to 697 working hours. During day shifts, residents spent 5.4 hours using a computer (mean total working time: 11.6 hours per day). On average, residents switched 15 times per hour from a task to another. Task-switching peaked between 8:00–9:00 and 16:00–17:00. Task-switching was not associated with resident’s characteristics and no association was found between task-switching and extra hours (Spearman r = 0.220, p = 0.137 for day and r = 0.483, p = 0.058 for evening shifts). Computer usage occurred more frequently at the beginning or ends of day shifts and was associated with decreased overall task-switching.

**Conclusion:**

Task-switching occurs very frequently during resident’s working day. Despite the fact that residents used a computer half of their working time, computer usage was associated with decreased task-switching. Whether frequent task-switches and computer usage impact the quality of patient care and resident’s work must be evaluated in further studies.

## Introduction

Frequent switches in resident’s ongoing tasks might not only have negative effects on patient safety because of the physical and cognitive workload and discontinuity in medical attention but might also impact resident’s capacity to organize a workday [1, 2].

Few quantitative studies focusing on frequency of task-switches occurring during resident’s work in general internal medicine have been published and comparisons are limited by different health systems and medical education programmes [1, 3].

Recent implementation of electronic health records (EHR) has dramatically changed medical ward organization [4]. Thus computer usage may not only have a negative effect on patient care or medical education [4, 5], but also influence patient-physician communication and satisfaction [6, 7]. While residents in internal medicine use EHR systems half of their working time [4], whether computer usage impacts residents’ workflow remains uncertain. A recent time motion study conducted in an ambulatory setting showed that physicians interacted with EHR during one third of the time they spent with patients [8], which differs from our hospital setting where residents in internal medicine seldom do so [9].

We therefore aimed: a) to observe the frequency of task-switches occurring during resident’s work, and more specifically during important medical tasks; b) to identify factors associated with task-switching; and c) to assess whether task-switching was associated with computer usage, in the general internal medicine division of a Swiss university hospital.

## Methods

In a large Swiss academic university hospital (www.chuv.ch), we conducted, between May 26 and July 24, 2015 a time-motion study to assess how residents in general internal medicine organize their working day. Our institution uses Soarian by Cerner^®^ EHR system. All residents (n = 36, % female = 64, median post-graduated training = 32 months [interquartile range 27–42]) rotating in medicine wards during the study period were included. The Human Research Ethics Committee of canton de Vaud certified that the study was exempt from human subject’s ethics review. All residents were informed of the study and signed a written consent. No patient identifier or health information was recorded. A detailed description of the study methods has been published elsewhere [9].

During day (08:00–18:00) or evening (16:30–23:00) shifts, trained external observers with a medical background collected, using a dedicated tablet application, resident’s tasks and whether these tasks were performed with or without a computer. Each task was recorded in real-time (in seconds). Two observations (i.e. two day shifts or one day and one evening shift) per resident were recorded.

Overall 22 different tasks were defined (S1 Table). We considered five tasks as medically important: 1) daily rounds of patients in charge (including review of the electronic medical records, test results, examination, communication, prescriptions, and orders); 2) giving or receiving handoff with the goal to transfer patient responsibility (including preparation of documents, participation to handoff meeting, and others exchanges of information, excluding supervision or exam request); 3) writing in EHR; 4) meetings families (including information exchange, explanation, and collection of opinions); 5) receiving or providing academic teaching (i.e. participation to conferences and attending rounds, self-study and paper review). Task-switching was defined as a switch from any task to another or from one medically important task to another and expressed as number of switches per hour.

Association between resident’s characteristics, shift type, computer usage and task-switching was assessed using a mixed model for repeated measures, adjusting for resident’s characteristics, shift type, and computer usage. Results are expressed as multivariate-adjusted mean task-switching rate ±standard error. Due to the skewed distribution of resident’s extra hours, the association between resident’s extra hours and task-switching was assessed by non-parametric Spearman correlation.

## Results

We observed 49 day and 17 evening shifts of 36 residents, amounting to 697 working hours. During day shifts, residents spent 45% of time performing important medical tasks and spent 5.4 hours using a computer (mean total working time: 11.6 ±1.3 hours per day).

**Fig 1:**
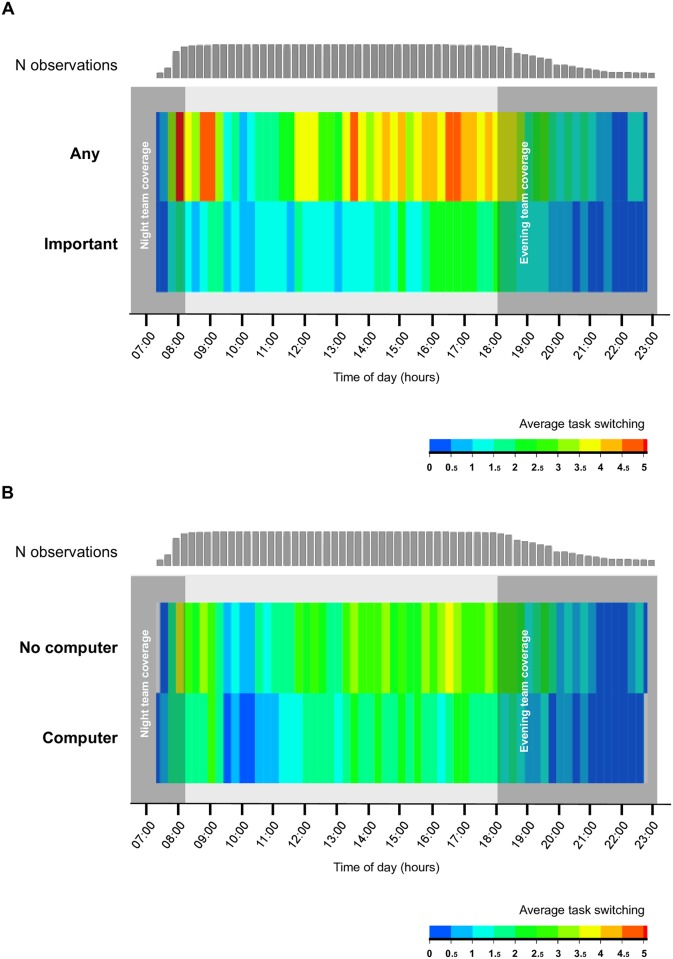
Average number of task-switching during day shifts. Panel A: By task category. Panel B: By computer usage. Time was split in 15-min periods; the black bars in the top row indicate the number of observations (range 1–49). ANY: switch from any task to another. IMPORTANT: switch from one medically important task (i.e. medical rounds, handoffs, writing in medical records, meetings with families and academic teaching received) to another.

On average, residents switched 15 times per hour from any task to another and six times per hour from one medically important task to another. Task-switching peaked between 8:00–9:00 and 16:00–17:00 (Fig 1). Residents switched more often from a medically important task to another during day shifts compared to evening shifts (7.0±0.3 vs. 5.3±0.6, p = 0.009).

Task-switching was not associated with resident’s characteristics and no association was found between task-switching and extra hours (Spearman r = 0.220, p = 0.137 for day and r = 0.483, p = 0.058 for evening shifts).

Computer usage occurred more frequently at the beginning or ends of day shifts and was statistically associated with decreased task-switching during any task, but not during important medical tasks. During evening shifts, computer usage was associated with a lower switching rate for both type of task categories (Table 1).

**Table 1:** Factors associated with hourly rate of task-switches, overall and stratifying by type of shift.

	TASK-SWITCHING^c^			
	During any^a^ task		During important ^b^ task	
		*P*-value		*P*-value
**Type of shift**				
Day (n = 49)	15.9 ± 0.6	0.245	7.0 ± 0.3	0.009^d^
Evening (n = 17)	14.7 ± 0.9		5.3 ± 0.6	
**Day shift (n = 49)**				
Gender				
Male	16.6 ± 0.9	0.276	7.2 ± 0.5	0.372
Female	15.3 ± 0.7		6.6 ± 0.4	
Post-graduated training				
≤32 months	15.1 ± 0.7	0.144	6.6 ± 0.4	0.400
>32 months	16.9 ± 0.9		7.2 ± 0.5	
Computer usage				
Yes	12.5 ± 0.7	<0.001^d^	6.7 ± 0.4	0.548
No	19.3 ± 0.7		7.0 ± 0.4	
**Evening shift (n = 17)**				
Gender				
Male	13.9 ± 2.5	0.722	4.2 ± 1.6	0.441
Female	14.8 ± 1.0		5.5 ± 0.6	
Post-graduated training				
≤32 months	15.3 ± 1.6	0.649	5.6 ± 1.0	0.832
>32 months	14.4 ± 1.2		5.3 ± 0.7	
Computer usage				
Yes	10.6 ± 1.1	<0.001^d^	4.4 ± 0.7	<0.001^d^
No	18.8 ± 1.1		6.4 ± 0.7	

^a^ANY: any switch, from one task to another.

^b^ IMPORTANT: switch from one important medical task (i.e. medical rounds, handoffs, writing in medical records, meetings with families and academic teaching received) to another task.

^c^Results are expressed as multivariate-adjusted hourly average ± standard error. Statistical analysis using a mixed model for repeated measures, adjusting for all variables in the table.

^d^Significant *P*-values

## Discussion

In a Swiss university hospital, general internal medicine residents switched tasks every four minutes on average, with peaks occurring in the morning and in the afternoon. The frequency of task-switching was two to three times lower during important medical tasks.

Two previous studies observing doctors working in medical and surgical wards in Australia and Germany, reported that doctors were interrupted on average 2.9 to 3.6 times per hours (i.e. every 15 or 20 minutes respectively) [1, 3]. Interruption was defined as the cessation or discontinuation of a current task, which covers only partially our definition of task-switching and may explain why we found such a high task-switching rate, occurring every 4 to 10 minutes, depending on the task.

Even if task-switching was lower during medically important tasks and had no impact on extra hours, whether task-switching is a prerequisite for efficient flexibility (defined as the capacity for a resident to adapt to changing working conditions and to adequately respond to multiple requests) remains uncertain [10]. Our findings highlight that residents are able to adapt their work according to task importance. Thus, residents may task-switch momentarily more often on purpose, ensuring a quick answer to healthcare needs, typically during day shifts compared to night shifts or during tasks of lower importance.

Interestingly, computer usage was associated with a lower task-switching rate, probably due to the fact that residents tend to work with the computer outside their duty hours in order to be more focused, or because medically important tasks, such as writing in medical records, require more concentration. Unfortunately, we could not assess whether a higher number of disruptions during working hours precluded computer use; or whether, while using the computer, residents did not shift tasks even if they could, and overcompensate during working hours. Still, the fact that residents use the computer more frequently before and after working hours (when there are less possibilities of disruption due to absence of family members or consultants) suggests that the first possibility is more likely.

Residents spend almost half of their working day with the computer, a finding also reported elsewhere [4]. Despite growing concerns that computer usage has a negative effect on patient care or medical education [4–7], our results show that computer usage is not associated with work disruption, and that residents seem to adapt their working time accordingly.

Our study has potential limitations. First, our study may be underpowered to find a statistical association between the factors studied and task-switching. However, to date, this is the largest existing time-motion study focusing on factors associated with task-switching. Second, the study was limited to a single specialty (i.e. general internal medicine) at one institution, which potentially limits generalization of our results to other settings. Thirdly, while we demonstrated that the rate of task-switching decreased during computer usage, it does not exclude the possibility that overall task-switching increased as residents attempted to complete more tasks when performing activities without computers.

However, our study has a major strength. We objectively assessed the frequency of task-switches based on the direct observation of resident’s at work and recorded each task in seconds. Thus, our data provide important and objective information for the medical education system.

## Conclusion

In conclusion, task-switching occurs very frequently during resident’s working day. Despite the fact that residents used computers half of their working time, computer usage was associated with decreased task switching. Whether frequent task-switches impact the quality of patient care and resident’s work must be evaluated in further studies.

## Supporting information

S1 Table Definitions of the 22 resident’s activities grouped in six categories. Activities of residents are exclusive. EHR = electronic health record.
(DOCX)
