# Supplementary material for: Computer usage and task-switching during resident’s working day: Disruptive or not?
Source: PLoS One. 2017 Feb 24;12(2):e0172878. doi: 10.1371/journal.pone.0172878 (PMC5325549; doi:10.1371/journal.pone.0172878)
Supplement: S1 Table — Activities of residents are exclusive. EHR = electronic health record. (DOCX) [file pone.0172878.s001.docx]

**S1 Table. This is the S1 Table Title. Definitions of the 22 resident’s tasks grouped in six categories**.

This is the S1 Table legend.Tasks of residents are exclusive. ^a^EHR = electronic health record

| **CATEGORY** | **TASK** | **DEFINITION** |
| --- | --- | --- |
| **Directly related to patients** | Admission | Admitting a new patient on the ward. Includes medical history, clinical examination, and communication. The activity starts with nominally looking after a new patient, i.e. reading the medical file or first contact with him. |
|  | Medical round | Daily round of patients in charge. Includes review of the EHR^a^ and test results, examination, communication, prescriptions, and orders. Daily sign-out round with the nurse is also included. |
|  | Discharge | Preparing patient discharge. Includes prescription writing, last interview, delivery and explanation of prescription. |
|  | Clinical procedures | Performing any medical procedure or test. Includes arterial blood gas, ascites paracentesis, puncture, chest tube withdrawal, central venous catheter, carotid bulb massage, and others. |
|  | Out of unit support | Attending the patient outside the ward. Includes oversight during exams, transfer to another department, and emergency situations. |
| **Communication** | News delivery | Announcing and discussing results, bad news or therapeutic orientation with need of a specific time. Includes therapeutic education. |
|  | Family meeting | Communicating with relatives or non-professional caregivers. Includes information exchange, explanation, and collection of opinions. |
| **Indirectly related to patients** | Looking for information | Looking for information. Includes looking in the paper record, EHR^a^, computer archives, or other support. Excludes activities related to admission. |
|  | Literature reviewing | Looking for scientific data to improve patient management. Includes medical textbooks, scientific papers, website of systematic review, online open search, and use of score calculation tools. |
|  | Writing in medical record | Writing notes, problem list, handoffs, or exams results in the EHR^a^. Excludes activities related to admission or discharge report. |
|  | Discharge summary | Producing medical reports. Includes brief report and discharge summary redaction or revision. |
|  | Handoffs | Giving or receiving handoff. Includes preparation of documents, participation to handoff meeting, and others exchanges of information. The goal is to transfer patient responsibility. Excludes supervision or exam request. |
|  | Supervision | Discussing with a senior physician with orientation to patient management. Includes patient's record review and short own patient presentation during attending rounds |
|  | Providers | Requesting information, specialized advice, or action from other providers. Includes general practitioner, specialist, and physiotherapist. Excludes relatives of the patient. |
|  | Patient administration | Completing administrative tasks for the patient. Includes appointments, x-ray prescription, specialized consultation demand. Excludes discharge summary redaction. |
|  | Multidisciplinary board | Attending to boards to discuss management of one or more patients. Includes specialized meeting, orientation meeting, team conflicts resolution or debriefing. Excludes meeting with patients or patient's family. |
| **Academic** | Training | Receiving training. Includes participation to conferences and attending rounds, self-study and paper review. Excludes literature reviewing. |
|  | Teaching | Providing teaching to others people: students, collaborators, nurses. |
|  | Research | Working for own clinical research or thesis. Excludes literature reviewing. |
| **Non-medical tasks** | Non-patient administration | Activity unrelated to a specific patient. i.e. answering professional emails. |
|  | Personal | Meeting his own needs. Includes food, toilets, private phone discussion, and private use of the computer. |
| **Transition** | Transition time | Transition to the next activity. Includes moving, hand washing, dressing, fetching or bringing something. |
